# Health service utilization, unmet healthcare needs, and the potential of telemedicine services among Korean expatriates

**DOI:** 10.1186/s12992-018-0433-y

**Published:** 2018-11-29

**Authors:** Ho Young Kim, Ju Young Kim, Hwa Yeon Park, Ji Hye Jun, Hye Yeon Koo, In Young Cho, Jinah Han, Yuliya Pak, Hyun Jung Baek, Ju Yeon Lee, Sung Hee Chang, Jung Hun Lee, Ji Soo Choe, Sun-kyung Yang, Kyung Chul Kim, Jeong Ha Park, Seul Ki Paik

**Affiliations:** 1 Department of Family Medicine, Seoul National University Hospital, Seoul, South Korea; 2 Department of Family Medicine, Seoul National University Bundang Hospital, Seongnam, South Korea; 3 Department of Family Medicine, Chamjoeun Hospital, Gwangju, South Korea

**Keywords:** Telemedicine, Health service utilization, Unmet health needs, Korean expatriates

## Abstract

**Background:**

With the significant growth of migration and expatriation, facilitated by increased global mobility, the number of Koreans living abroad as of 2016 is approximately 7.4 million (15% of the Korean population). Healthcare utilization or health problems, especially among expatriates in developing countries, have not been well researched despite the various health risks these individuals are exposed to. Consequently, we identified the health utilization patterns and healthcare needs among Korean expatriates in Vietnam, Cambodia, and Uzbekistan.

**Methods:**

This cross-sectional survey examined 429 Korean expatriates living in Vietnam (*n* = 208), Cambodia (*n* = 60), and Uzbekistan (*n* = 161) who had access to the Internet and were living abroad for at least 6 months. A 67-item questionnaire was used, and feedback was received via an online survey program. Stepwise logistic regression analyses were performed to evaluate factors associated with unmet healthcare needs and preferences of certain type of telemedicine.

**Results:**

We found that 45.5% (195/429) of respondents had used medical services in their country of stay. Among those who visited health institutions > 3 times, the most popular choice was general hospitals (39.4%, 15/38); however, they initially visited Korean doctors’ or local doctors’ offices. The most essential criteria for healthcare service facilities was a “skilled professional” (39.3%, 169/429), 42% wanted a health program for chronic disease management, and 30% wanted specialized internal medicine. A substantial number wanted to access telemedicine services and were willing to pay for this service. They were particularly interested in experts’ second opinion (61.5%, 264/429) and quick, 24-h medical consultations (60.8%, 261/429). Having unmet healthcare needs and being younger was strongly associated with all types of telemedicine networks.

**Conclusions:**

Nearly half of the expatriates in developing countries had unmet healthcare needs. Telemedicine is one potential solution to meet these needs, especially in developing countries.

## Background

In 2017, more than 258 million people crossed the borders [1]. The globalized world economy makes global mobility and flexibility in the workforce very rapid; therefore, migration and expatriation is likely to grow substantially [2]. As of 2016, the number of Koreans living abroad is approximately 7.4 million (15% of the Korean population) [3]. Despite this phenomenon, healthcare utilization and health problems among expatriates have been insufficiently researched. Specifically, expatriates living in developing countries are a vulnerable population who face several health problems including acute infectious diseases; unsafe water; and problems associated with adjusting to climate, stress, and cultural/language barriers [4].

There is no widely adopted definition of an ‘expatriate’ that differs from a ‘traveller’; however, in general, an expatriate is an individual who moves to another country, thus changing the dominant place of residence (i.e., where the person’s family lives, where the person’s economic interests are, where the person spends more than 183 days/year, or the person’s nationality as in ordered criteria) and obtaining legal work abroad [5]. Therefore, the expatriate has migrant status.

Regarding *self-initiated expatriation*, the decision to move internationally is solely made by the individual who initiates the expatriation. However, *assigned expatriation* denotes an employee who is sent abroad by his/her company, usually receiving an expatriate contract [6]. Notably, 63% expatriates are individual workers, 14% are students, 8% are retired expatriates, and 3% are corporate transferees [2], suggesting that self-initiated expatriates are the majority, and this proportion is expected to grow in the future.

A study examining Portuguese expatriates’ health reported that one third of expatriates experienced significant psychological stress. Further, 20% reported new health problems and the need for medical assistance, 5% were hospitalised, and 64% reported general psychological symptoms [7, 8]. The most significant health risks to expatriates were accidents (especially road traffic accidents), mugging, and kidnapping; however, they also experienced injuries due to extreme climates or poor air quality. Expatriates are also vulnerable to risky drinking, risky sexual behaviours, adjustment disorders, and cultural/language barriers; in addition, facility/job factors also affect their physiological or psychological wellbeing [9, 10]. For example, if expatriates have a chronic disease such as diabetes or asthma, it can be difficult to treat due to inadequate medical infrastructures [11].

Recently, the Korean government started a government-funded pilot telemedicine programme for Korean expatriates, which involves providing healthcare safety for their own citizens who live in resource-limited areas. [12] To design and implement an effective telehealth network system for Korean expatriates, their health-related risk factors and health utilization pattern should be investigated. Consequently, we identified the health service utilization patterns and needs assessment as well as investigated potential types of telemedicine for health issues among Korean expatriates and their partners living in Vietnam, Cambodia, and Uzbekistan.

## Methods

### Study design

This study was part of the project of “Development and demonstration of telehealth counseling program for overseas Koreans” funded by the Korea Health Industry Development Institute. Detailed project with its outcomes were described elsewhere [12, 13] but briefly speaking, we set up global Digital Health Centers(DHCs) for overseas Korean populations in Vietnam, Uzbekistan and Cambodia. These sites were chosen based on the location of Korean Association centers because their help with support was needed in establishing DHCs and also in relation to the location of collaborating hospitals with Seoul National University Bundang Hospital.

Korean nurses, who were also expatriates, were recruited as coordinators and worked in each digital healthcare centre—helping Korean expatriates using telehealth counselling service to connect with Korean doctors.

The three south Asian countries have different status in adoption of national health system: The healthcare system has a mixture of public and private provision in Vietnam with 77% of the population covered under universal coverage in 2015 [14]. Cambodia has a mixed service delivery system. Public health service delivery is organized through two levels of services, health center and referral hospitals. Private practitioners, workplaces and international NGOs deliver a limited range of services. But lack of resources, expertise, services, logistics, data as well as lack of regulation and monitoring to support the prevention and management of chronic disease limited the adequate health service deliveries in both Cambodian people as well as expatriates [15]. Uzbekistan’ health system consists of three distinct hierarchical layers: the national, regional and local level made up of rural districts or cities, with a relatively small private sector. Government has significantly limited the type of services that can be provided in the private sector, in particular with regard to complex surgical procedures [16].

There are not many literatures or regulations on foreigners or expatriates living in those countries but in general health services are covered by work-sponsored health insurance if they are expatriated for their work. Individual workers or students who don’t belong to certain organization might have limited access to health care services and don’t have adequate insurance coverage as well. That is why Korean government saw the expatriates as vulnerable population to health service and funded telehealth counseling program.

We employed a cross-sectional survey to determine the health service utilization and the healthcare needs of Korean expatriates who were living in Vietnam, Cambodia, or Uzbekistan. All participants had Internet access and were living in the new country for at least 6 months.

### Participants

Participants were 429 Korean expatriates living in Vietnam (*n* = 209), Cambodia (*n* = 60), or Uzbekistan (*n* = 181). Participants were aged > 19 years and had been living in the corresponding countries for at least 6 months and were chosen through stratified sampling based on the sex and age of the population registered as Korean expatriates in each country. Participants were recruited from announcements at overseas Korean Associations or newspaper advertisements from a Korean–Vietnam news company in Ho Chi Minh city.

### Data collection

A structured questionnaire was developed based on the review of relevant literature about medical service utilization, a focus group interview concerning expatriates’ healthcare needs [13], and expert-group consultations.

The questionnaires consisted of demographic information and health service utilization in the past 6 months from drug stores, outpatient clinic visits, emergency room visits, and hospital admissions. Questionnaires also addressed reasons for choosing the specific types of healthcare services categorized as International Classification of Disease 10th Codes (ICD-10) and types of preventive services the expatriates wanted.

Respondents were asked about what types of healthcare services they wish to be provided with when consulting Korean doctors online. These healthcare service types were derived from qualitative interviews with Korean expatriates [13] and included having an online family doctor for a regular check-up or health consultation, a 24-h online medical consultation whenever needed, receiving a second opinion from Korean doctors regarding their medical status, and telehealth diagnosis with treatment if feasible for dermatologic or psychiatric problems. Participants responded using a 5-point Likert scale (1–5), depending on the strength of the need. Respondents were also asked about willingness to pay for the service; they were instructed to indicate the amount in their local currency. The prices were then converted into US dollars; then, interquartile and median values were calculated.

A 67-item questionnaire was then distributed, and feedback was received via an Internet survey programme in June 2017. We used SurveyMonkey®, a popular online survey development cloud-based software programme. Participants were enlisted through expatriate communities and local expatriate news media by sharing the URL of the online survey programme through emails and social network groups.

If participants did not respond to the online survey, two reminders (1 week apart) were emailed to participants. However, 49 people aged > 60 years (11.4% of the study population) were visited by the coordinators and provided with assistance so they could complete the survey digitally.

### Data analyses

We performed a descriptive analysis. Participants’ baseline characteristics are represented with frequencies and percentages. The feedback on the questions about healthcare utilization and unmet healthcare needs was analysed  for each of the three countries using a one-way analysis of variance. However, when there were no significant differences noted among the countries, the results were aggregated to obtain a larger sample size.

The chi-square or Fisher’s exact test in case of small numbers was performed between men and women in categorical variables of healthcare service utilization. Stepwise logistic regression analyses were performed to evaluate factors associated with unmet healthcare needs and preferences of certain type of telemedicine. *P*-values < .05 were considered significant. All analyses were performed using STATA 13 (StataCorp., College Station, TX, USA).

## Results

Participants’ demographic characteristics are shown in Table 1. Further, the 67-item questionnaire survey was broadly divided into three categories: current health service utilization, unmet healthcare needs, and potential types and preference of telemedicine for fulfilling unmet needs.

**Table 1:** Participants’ baseline characteristics (*N* = 429)

	Total	%
Country		
Vietnam	208	48.5
Uzbekistan	161	37.5
Cambodia	60	14.0
Sex		
Men	229	53.4
Women	200	46.6
Age (years)		
20–29	48	11.2
30–39	81	18.9
40–49	194	45.2
50–59	57	13.3
60–69	39	9.1
≥ 70	10	2.3
Marital status		
Never married	74	17.3
Married	336	78.5
Divorced/separated/widowed	18	4.2
Education		
≤ high school	80	18.6
≥ university	340	79.3
Job		
Licensed professional	17	4.0
Administration	134	31.4
Service/engineer	28	6.6
Homemaker	121	28.3
Student	20	4.7
Self-employed	73	17.1
Other	34	8.0
Access time to hospital (minutes)		
< 30	314	73.7
30_60	89	20.9
60–90	17	4.0
≥ 90	6	1.4
Monthly income (USD)		
< 1000	41	9.8
1000–3000	53	12.6
3000–5000	86	20.5
5000–7000	109	26.0
≥ 7000	131	30.5
No response	9	2.1
Medical insurance		
Employer-based	154	36.0
Private (covering overall cost)	108	25.2
Private (covering individual visit)	136	31.8
None	114	26.6

### Healthcare service utilization

The survey assessed the use of outpatient services, pharmacies, emergency rooms, and hospital admission of Korean expatriates. The respondents were asked to answer whether they had used the services over the last 6 months and to choose multiple reasons for requiring the services (Table 2). We identified the patterns of expatriates’ choice of healthcare institutions and the factors affecting their choice among those who had ever used medical services in their country of stay (*N* = 195). We also compared healthcare service utilization between men (*N* = 229) and women (*N* = 200). Respiratory symptoms consisted of most common reasons in outpatient clinic in both men and women, followed by musculoskeletal condition and gastrointestinal disease. Of note, disease of circulatory system such as hypertension or angina occurred more frequently in men. (*P*-value = 0.0094).

**Table 2:** Healthcare service utilization among Korean expatriates (*N* = 429)

	Men, *n* (%) (*N* = 229)	Women, *n* (%) (*N* = 200)	*P*-value
Number of expatriates who ever visited a local outpatient clinic	100 (43.7)	94 (47.0)	0.5150
Reasons for seeking outpatient care (ICD-10 codes)			
Diseases of the respiratory system	32 (14.0)	43 (21.5)	0.0406
Diseases of the musculoskeletal system and connective tissue	19 (8.3)	19 (9.5)	0.6618
Diseases of the circulatory system	17 (7.4)	4 (2.0)	0.0094
Diseases of the digestive system	14 (6.1)	16 (8.0)	0.4447
Others^a^	65(28.4)	63 (31.5)	0.3129
Number of expatriates who ever visited a local pharmacy	147(65.3)	148 (75.1)	0.0286
Reasons for a pharmacy visit			
Prescription medicine	35 (15.3)	33 (16.5)	0.7308
Non-prescription medicine	94 (41.0)	104 (52.0)	0.0232
Non-medical products	42 (18.3)	50 (25.0)	0.0937
Number of expatriates who ever visited a local emergency department	10 (4.4)	10 (5.0)	0.7639
Reasons for an emergency department visit (ICD-10 codes)			
Injury, poisoning and certain other consequences of external causes	3 (1.3)	1 (0.5)	0.2839
Diseases of the respiratory system	2 (0.9)	1 (0.5)	0.3996
Certain infectious and parasitic diseases	1 (0.4)	2 (1.0)	0.3487
Diseases of the digestive system	2 (0.9)	1 (0.5)	0.3996
Others^b^	5 (2.2)	5 (2.5)	0.4989
Number of expatriates who ever admitted to a local hospital	13 (5.7)	5 (2.6)	0.1110
Reasons for admission visit (ICD-10 codes)			
Diseases of the digestive system	4 (1.7)	1 (0.5)	0.1887
Certain infectious and parasitic diseases	3 (1.3)	1 (0.5)	0.2839
Diseases of the respiratory system	1 (0.4)	1 (0.5)	0.4989
Others^c^	6 (2.6)	2 (1.0)	0.2839

^a^Other reason for outpatient visit: 25 skin conditions, 15 endocrine, nutritional and metabolic diseases, 14 neurologic conditions, 11 infectious diseases, 10 eye conditions, 10 urologic conditions, 9 ear diseases, 4 pregnancy, 3 accidents, 1 neoplasm, 1 psychiatric condition, and 12 dental problems

^b^Other reason for emergency department visit: 2 neurologic conditions, 2 cardiac problems, 1 pregnancy, 1 urologic condition and 1 musculoskeletal condition

^c^Other reason for admission to hospital: 2 endocrine, nutritional and metabolic diseases, 2 respiratory conditions and 2 pregnancies

Accident was the common reason of utilization for an emergency department visit, while gastrointestinal disease such as diarrhea was the common reason of admission to the hospital.

For the initial visit, the most popular choice was Korean and local doctors’ offices equally (ns = 46), except for Uzbekistan, where there were no available Korean doctors’ offices. Approximately half used different medical service again. For their second visit, however, it was noted that their preferences for health institutions had slightly changed. Korean doctors were still the most popular choice for their second visit and became more popular (30.7% vs. 23.5%). Local doctors lost ground to a certain degree (19.7% vs. 23.5%). Most notable was that people tended to visit general hospitals the second time (28.5%), which was a significant increase since the first time (14.8%). Even after the second visit, 19.4% expatriates were not satisfied and required additional medical service use. For their third visit, preference for general hospitals became even stronger, far ahead of primary Korean and local doctors’ care (39.4% of total visits).

We subsequently examined factors associated with choosing a healthcare provider. The three most crucial factors were ‘skilled medical professionals’ (39.3%, 169/429), ‘specialised medical departments’ (21.6%, 93/429), and ‘Korean doctors’ (11.5%, 50/429).

### Unmet healthcare needs among Korean expatriates

We inquired about the medical departments and health programmes that the expatriates wished to have available in their resident countries. Respondents were given 16 choices of medical departments and asked to prioritise the three medical departments that they needed the most. The largest number of respondents (29.8%, 128/429) wished to have an internal medicine department, followed by family medicine, paediatrics, neurosurgery, and obstetrics & gynaecology, respectively. The most commonly needed healthcare programmes among the three countries were chronic disease management (42.1%, 181/429), followed by education about emergency care (17.2%, 74/429) and child/adolescent care (10.2%, 42/429). Other healthcare programme needs included stress management and education for cardiopulmonary resuscitation methods (6.2%). Overall, 178 participants (41.5%) had unmet healthcare needs. Being a woman (OR = 2.21, 95% CI = 1.39–3.52), having private insurance (OR = 1.21, 95% CI = 1.01–1.44), and having arthritis (OR = 2.82, 95% CI = 1.08–7.41) were associated with unmet healthcare needs.

### Potential types and preference of telemedicine for providing unmet healthcare needs

According to the focus group interview [16], we extracted four types of telemedicine programmes people wished to have in the future (see Table 3). We also looked at several factors associated with potential preferential type of telemedicine program (Table 4).

**Table 3:** Potential types and preferences of telemedicine and willingness to pay (*N* = 429)

Family doctor			
Pay scheme	Preference, Mean (SD)	*n* (%)	Expected price median (IQR)
Total	3.6 (0.9)	230 (53.6)	
Annually		80 (34. 8)	61.5 (28.0, 123.0)
Monthly		29 (12.6)	18. 5 (12.3, 35.2)
As needed		121 (52.6)	13.2 (6.6, 26.4)
A quick, 24-h medical consultation			
Pay scheme	Preference, Mean (SD)	*n* (%)	Expected price median (IQR)
Total	3.7 (0.9)	261 (60.8)	
Annually		56 (21. 5)	44 (23.9, 88.0)
Monthly		15 (5.7)	13.2 (9.9, 23.3)
As needed		190 (72.7)	8.8 (5.0, 19.6)
Second opinion			
	Preference, Mean (SD)	*n* (%)	Expected price median (IQR)
Total	3.6 (0.8)	264 (61.5)	
As needed			12.7 (8.8, 22.0)
Tele-diagnosis with prescription			
Pay scheme	Preference, Mean (SD)	*n* (%)	Expected price median (IQR)
Total	3.4 (0.8)	204 (47.5)	
Annually		47 (23.0)	36.9 (21.0, 88.0)
Monthly		20 (9.8)	13.2 (12.3, 22.0)
As needed		137 (67.2)	10 (6.2, 22.0)

Note. *SD* standard deviation;*IQR* interquartile range

**Table 4:** Factors associated with preferences of types in telemedicine among expatriates

Factors	Odds ratio	95% confidence interval
	Having a family doctor	
Having unmet healthcare needs	2.8	(1.9, 4.1)
Being married	2.8	(1.8, 4.4)
Having hypertension	2.2	(1.2, 3.8)
	A quick 24-h consultation	
Having unmet healthcare needs	2.2	(1.5, 3.3)
Older age by 10 year difference	0.8	(0.6, 0.9)
Higher self-rated health	1.2	(1.0, 1.3)
Being married	1.9	(1.2, 3.1)
Higher income	1.2	(1.0, 1.4)
Having hypertension	2.2	(1.2, 3.9)
	Second opinion	
Having unmet healthcare needs	1.7	(1.2, 2.6)
Higher education	1.4	(1.0, 1.9)
	Tele-diagnosis with prescription	
Having unmet healthcare needs	1.8	(1.2, 2.7)
Older age by 10 year difference	0.7	(0.6, 0.9)
Higher self-rated health	1.2	(1.0, 1.4)
Being married	1.8	(1.1, 3.0)
Having diabetes mellitus	4.1	(1.8, 9.4)

## Discussion

To our knowledge, this was the first report of health service utilization patterns among Korean expatriates, which provides a glimpse of the unmet health needs and how to conceptualise and provide healthcare service including telemedicine across the border. The three most common reasons for seeking healthcare services were respiratory conditions for outpatient clinics, gastrointestinal conditions for admission, and accidents for emergency room visit. Similar findings were reported among Portuguese expatriates’ health, where new health problems occurred in 25%, and the most common was acute respiratory infection [7].

Further, the language barrier was a significant factor in selecting hospital type.

Communication between healthcare providers and patients is critical; however, language barriers and cultural differences are obstacles to effective communication [17]. Expatriates tended to reduce this type of miscommunication by selecting Korean doctors’ offices, especially in Vietnam or Cambodia; however, when their problems went unresolved, they sought professional healthcare providers from secondary or tertiary hospital, even when they were already crowded [18]. Consequently, telemedicine has vast potential in reducing communication and space barriers.

Notably, nearly half of the expatriates in this study could not access healthcare services although they had health problems. Being female, having private insurance, and having arthritis were associated with unmet healthcare needs. Considering that one third of study participants were homemakers aged in their 30s or 40s, they might find it difficult to obtain reliable obstetric or gynaecologic treatment. There may also be a gap between what participants pay for their insurance, their expectations, and the actual healthcare they receive. Finally, having a chronic disease such as arthritis may require long-term treatment from a multidisciplinary team [19], which can be difficult for expatriates to ascertain. Targeting the needs of these populations should be a goal of telemedicine systems.

Moreover, telemedicine is essential when distance is a critical factor in accessing qualified healthcare services [20]. Most telemedicine projects in developing countries involve remote diagnoses and education [21]. The success rate strongly depends on the hardware prerequisites and telecommunication requirements, which are fulfilled more effectively with low costs in urban (vs. rural) areas.

The average medical price for an expatriate to visit an outpatient clinic once was approximately 15–20 USD. Given that outpatient consult for general symptoms such as common cold symptoms costs approximately 20 USD in these countries, the surveyed expatriates may have set this average price for telehealth at 75–100% of the general medical consultation. However, as no specific telehealth programmes have been demonstrated, such feedback should be received simply to reflect acceptability and feasibility of telehealth services among expatriates. The specific price of 15–20 USD could hardly be used as an absolute standard and it is essential to remember that the fee for a medical consultation differs per the healthcare costs in each country.

Expatriates were particularly interested in second medical opinion services and 24-h medical consultations. Regardless of hospital type, expatriates with unmet healthcare needs wanted to be connected through telemedicine with Korean doctors. Further, younger participants preferred all types of telemedicine systems, and those with diabetes wished to get prescriptions through telemedicine.

We also performed the analysis of associated factors with preferences of telemedicine counselling types among sub-group of expatriates who visited outpatient clinic more than second time or changed their hospital from private clinic to general hospital or tertiary hospital. The most strongly associated single factors with telemedicine counselling was having unmet healthcare needs.

### Limitations

This study had some limitations. We surveyed Korean expatriates living in Vietnam, Cambodia, and Uzbekistan; however, most were from Vietnam, which, as of 2017, has roughly 124,500 Korean expatriates [3]. By comparison, around 10,000 Korean expatriates live in Cambodia, which was less represented in this study. Although we addressed common health issues, needs and service utilization among Korean expatriates living in developing countries, our results are not generalizable to expatriates from other countries.

Recruiting study participants through an online survey could be challenging especially in developing countries where the Internet is unstable or is strictly controlled by the government. However, in this study, we showed that it was feasible to recruit study participants through an online e-mail system with the help of local Korean associations. Soon, online-based survey or app-based research will be expanding into global populations, overcoming time and distance barriers, and replacing traditional surveys.

## Conclusions

Expatriates in developing countries experience diverse health problems, and nearly half of them have unmet healthcare needs. Nearly half also visited an outpatient clinic at least once, most frequently due to respiratory infection. Most preferred telemedicine programmes that included obtaining a second medical opinion from experts and a quick, 24-h medical consultation. Having unmet healthcare needs and being younger were strongly associated with all types of telemedicine networks. Telemedicine is a potential solution to solve the unmet healthcare needs among expatriates living in developing countries.
